# Comparative proteomic analysis of maize (*Zea mays* L.) seedlings under rice black-streaked dwarf virus infection

**DOI:** 10.1186/s12870-018-1419-x

**Published:** 2018-09-12

**Authors:** Runqing Yue, Caixia Lu, Xiaohua Han, Shulei Guo, Shufeng Yan, Lu Liu, Xiaolei Fu, Nana Chen, Xinhai Guo, Haifeng Chi, Shuanggui Tie

**Affiliations:** 1 Henan Academy of Agricultural Sciences, Zhengzhou, China; 2 The Henan Provincial Key Laboratory of Maize Biology, Zhengzhou, China

**Keywords:** Maize rough dwarf disease, Rice black streaked dwarf virus, Differentially accumulated proteins, Metabolism, Maize

## Abstract

**Background:**

Maize rough dwarf disease (MRDD) is a severe disease that has been occurring frequently in southern China and many other Asian countries. MRDD is caused by the infection of *Rice black streaked dwarf virus* (RBSDV) and leads to significant economic losses in maize production. To well understand the destructive effects of RBSDV infection on maize growth, comparative proteomic analyses of maize seedlings under RBSDV infection was performed using an integrated approach involving LC-MS/MS and Tandem Mass Tag (TMT) labeling.

**Results:**

In total, 7615 maize proteins, 6319 of which were quantified. A total of 116 differentially accumulated proteins (DAPs) were identified, including 35 up- and 81 down-regulated proteins under the RBSDV infection. Enrichment analysis showed that the DAPs were most strongly associated with cyanoamino acid metabolism, protein processing in ER, and ribosome-related pathways. Two sulfur metabolism-related proteins were significantly reduced, indicating that sulfur may participate in the resistance against RBSDV infection. Furthermore, 15 DAPs involved in six metabolic pathways were identified in maize under the RBSDV infection.

**Conclusions:**

Our data revealed that the responses of maize to RBSDV infection were controlled by various metabolic pathways.

**Electronic supplementary material:**

The online version of this article (10.1186/s12870-018-1419-x) contains supplementary material, which is available to authorized users.

## Background

Maize (*Zea mays* L.) is a large shared food and energy crop globally. However, the quality as well as production of maize always encounter several serious threats in the form of viral diseases, such as maize rough dwarf disease (MRDD) [1]. MRDD, a widespread and destructive disease with dwarfing symptoms, is mainly caused by the infections of *rice black streaked dwarf virus* (RBSDV) in China, *maize rough dwarf virus* (MRDV) in Europe, and *Mal de Rio Cuarto virus* in South America [2, 3]. RBSDV, with similar genome structures to MRDV, is a member of the genus *Fijivirus* and the family Reoviridae [2]. The spread of RBSDV among graminaceous hosts, such as rice, maize, wheat and barley, is dependent on brown planthoppers in a persistent and propagative manner [4].

RBSDV contains 10 genomic double-stranded RNA segments, named from S1 to S10, encoding at least 13 proteins [5]. In rice and maize, the pathogenic pathways have been partially elucidated. For example, the N-terminal part of the P5–2 protein is required for binding to chloroplasts [6]. Another protein of RBSDV, P7–2, functions as a key subunit of Skp1/Cul1/F-box complex ubiquitin ligase by interacting with SKP1 [7]. In rice and maize, the P7–2 protein is also involved in gibberellin pathway by interacting with GID2 during the RBSDV infection [8]. P9–1 of RSBSDV is an alpha-helical protein that plays important roles in the early phase of virus life cycle by forming intracellular viroplasms [9].

Some studies have reported a series of proteins, genes, and small RNAs that are responsive to the RBSDV infection under various natural conditions. Gene expression profile analyses showed that 4099 genes, including a number of stress response- and development-related genes were altered significantly by the RBSDV infection in maize [10]. Expression of several calmodulin-binding transcription activator (CAMTA) genes significantly changed under RBSDV infection, suggesting a role of CAMTA-mediated Ca^2+^ signaling in maize susceptibility to virus infection [11].

The involvement of transcription factors under the RBSDV infection has also been revealed. In rice, 75 members of the NAC family were differentially accumulated during the infections of RBSDV and other viruses [12]. Dual transcriptome analysis revealed a total of 28 differential expressed genes (DEGs) and 1085 DEGs at 1.5 day and 3 day post-artificial inoculation with RBSDV, respectively [13]. Transcriptional changes of a susceptible rice cultivar in the responses to RBSDV have also been analyzed at two time points [14].

Identification of DAPs using mass spectrometry (MS)-based peptide mass fingerprinting and tandem MS/MS-based peptide sequencing is a popular technique for protein quantification [15]. Although several proteome-based works in maize have been conducted, very few proteomic datasets on the response to RBSDV infection have been published [16]. In rice, cross-talk between the responses to H_2_O_2_ treatment and to RBSDV infection suggested pivotal roles of H_2_O_2_ in the responses to RBSDV infection [17]. Recently, a label-free quantitative proteomic analysis showed that chitosan oligosaccharide enhanced the resistance of rice to RBSDV infection by activating the mitogen-activated protein kinase signaling cascade pathway [18]. To investigate the responses of maize seedlings to RBSDV infection at the proteome level, comparative proteomic analysis was performed.

## Methods

### Plant material and RBSDV inoculation

Maize, *Zea mays* L. inbred line B73, seeds were surface washed, soaked in clean H_2_O for 2 h, and then germinated in an incubator at 28 °C overnight. Seedlings were grown in a greenhouse with a photoperiod of 16 h light/8 h dark, relative humidity of 58%, and light intensity of 120 μmolm^− 2^ s^− 1^. The liquid Hoagland medium at pH 5.8 was used as nutrient solution. Two-week-old seedlings at the third leaf stage were used for RBSDV inoculation.

Adult planthoppers were obtained from wheat seedlings in open areas in spring 2015, in Zhengzhou, China. These adult planthoppers were transferred to a growth cabinet and the growth conditions were set as 26 ± 2 °C with a 14 h light to 10 h dark photoperiod and relative humidity of 68%. Newly hatched first instar nymphs were collected by cage after **3** weeks. Fresh wheat seedlings were used to rear these nymphs every **3** days. Adult planthoppers were allowed to lay eggs on wheat seedlings for 2 d to generate nymphs at similar development stage. The presence of RBSDV in wheat seedlings were confirmed by enzyme linked immunosorbent assay [19].

RBSDV inoculation was performed as in our previous work [11]. Briefly, viruliferous planthoppers were obtained from instar nymphs for 5 h and were released to maize seedlings (100 planthoppers for each seedling) for a 3-day acquisition access period. The planthoppers were transferred to new wheat seedlings for an incubation period of 25 d in a greenhouse. Then, about 10 viruliferous adult planthoppers for each seedling were put on maize seedlings for a 6-day inoculation access period. A separated group of seedlings inoculated with health planthoppers was prepared to be used as a control group.

### Calculation of RBSDV copy number

Total RNAs were isolated from maize seedlings using a RNeasy plant mini kit (Qiagen, Hilden, Germany) according to its protocol. DNA contamination in total RNAs was removed by DNase I application (Sangon, Shanghai, China). Based on the S6 segment of RBSDV, two primers: TCAGCAAAAGGTAAAGGAACG (P1) and AGAGCTCTTCTAGTTATTGCG (P2), were designed for qRT-PCR. The PCR products were used as standard. The standard DNA solution was serially diluted 10-fold to obtain a gradient of DNA concentrations for standard curve generation. For RBSDV copy number determination, the Ct value was compared with the values on the standard curve.

### Sampling

The aboveground part of seedlings, not a whole seedling, was used for protein extraction. Five randomly selected seedlings were treated as a one biological replicate. In our experiment, three biological replicates were used for the control and the infected groups, respectively.

### Protein extraction and trypsin digestion

Samples from maize seedlings were selected and ground with liquid N2. Tissue powder was then transferred to a new 1.5 mL tube and sonicated on ice in lysis buffer containing 8 M urea, 2 mM Ethylenediaminetetraacetic acid, 10 nM dithiothreitol and 1% protease inhibitor Cocktail (P8849, Sigma-Aldrich, Beijing, China). The remaining debris was removed by centrifugation at 20,000 g at 4 °C for 10 min. The protein was precipitated with cold 15% TCA for 4 h at − 20 °C. After centrifugation at 4 °C for 3 min, the supernatant was discarded. Protein sample was redissolved in buffer containing 8 M urea, 100 mM tetraethyl-ammonium bromide Triethylammonium bicarbonate (pH 8.0), and its concentration was measured by a commercial protein assay (PTM, Hangzhou, China).

The protein solution was reduced with 10 mM dithiothreitol for 1 h at 37 °C and alkylated with 20 mM iodoacetamide for 45 min at room temperature in darkness. For trypsin digestion, protein samples were diluted by adding 200 mM TEAB buffer to bring the final urea concentration under 2 M. The proteins samples were digested with trypsin at a ratio of 1:50 (mass ratio, trypsin: protein) for the first round of digestion overnight and at a ratio of 1:100 (mass ratio, trypsin: protein) for the second 4 h round of digestion. The digested samples were vacuum dried. About 100 μg of each protein sample was digested by trypsin.

### TMT labeling and HPLC fractionation

Sample peptides were desalted using a Strata X C18 SPE column (Phenomenex, Torrance, US) and were dried by vacuum. Resulting peptides were reconstituted using a six-plex TMT kit (ThermoFisher, Shanghai, China) according to its operation manual. One unit of TMTsixplex™ Isobaric Label Reagent was used to treat 100 μg of peptide samples. One unit of reagent was mixed with 41 μL of acetonitrile on the rotator for 5 min at room temperature. TMT reagent mixture was added to peptide samples and was incubated at 25 °C for 2 h. Then, the reaction was terminated by application of 5% hydroxylamine, and the labeled peptides were combined and vacuum dried.

The sample peptides were fractionated using alkaline reverse-phase HPLC with an Agilent 300 Extend C18 column (Agilent, Shanghai, China). Briefly, sample peptides were fractionated with a gradient of 2–60% acetonitrile in 10 mM ammonium bicarbonate during 80 min into 80 fractions (pH 10). All fractions were combined into 18 fractions and vacuum dried by centrifugation.

### LC-MS/MS analysis

Peptides were firstly dissolved in 0.1% formic acid (FA) solution and were loaded onto an Acclaim PepMap 100 reversed-phase pre-column (ThermoFisher, Shanghai, China). Separation of peptides was performed using an Acclaim PepMap RSLC reversed-phase analytical column (ThermoFisher, Shanghai, China) on an EASY-nLC 1000 UPLC system. The gradient of solvent B (0.1% FA in 98% acetonitrile (ACN)) was increasing from 6 to 25% during 26 min, increasing from 25 to 40% during 8 min, climbing to 80% within 3 min, and holding at 80% for 3 min with a steady flow rate of 400 nL/min. After equilibration, the resulting peptides were determined by Q ExactiveTM hybrid quadrupole-Orbitrap mass spectrometer (ThermoFisher, Shanghai, China).

MS data were acquired by a series of cyclic scans at a high resolution of 70,000 using a data-dependent acquisition procedure, followed by scans at a relative low resolution of 17,500. The top 20 intense ions were determined under a threshold ion count of 1e4 in the MS survey scan with 30.0 s dynamic exclusion. The electrospray voltage was set at 2.0 kV and 5e4 ions were accumulated for MS/MS spectra generation. The saw mass spectrometry proteomics data have been deposited to the Proteome EXchange Consortium via the PRIDE partner repository with the dataset identifier PXD008186.

The quantitative value of the unique peptide was calculated according to the ratio of the ion signal intensity in MS2 of the TMT reporter ions. For TMT quantification, the ratios of the TMT reporter ion intensities in MS/MS spectra (m/z 126–131) from raw data sets were used to calculate fold changes between samples. For each sample, the quantification was mean-normalized at peptide level to center the distribution of quantitative values. Protein quantitation was then calculated as the median ratio of corresponding unique peptides for a given protein. Two-sample, two-sided t-tests were used to compare expression of proteins. In general, a significance level of 0.05 was used for statistical testing, and we reported the *P* value or significance level any time a statistical test was performed.

### Database search

The MS/MS data were searched against the MaizeGDB v4 (https://www.maizegdb.org/) and Maize SwissProt database using Andromeda with integrated MaxQuant search engine (v.1.5.2.8) with default parameters. The maximum allowable mass error was 10 ppm for precursor ions and 0.02 Da for fragment ions. Carbamido-methylation on Cys was selected as invariable modification and oxidation on Met was set as variable modification. False discovery rate (FDR) thresholds for the identification of peptides and proteins were specified at lower than 1% and the minimum length of peptide was seven amino acids. A TMT-6-plex kit was used for quantification of the resulting peptides. The quantitative level of peptide was measured according to its ion signal intensity ratio in the secondary spectrum.

### Protein annotation

For Gene Ontology (GO) annotation, a reference proteome was derived from the UniProt-GPA database. IDs of all identified proteins were converted to the UniProt IDs, and the proteins were mapped onto the reference proteome. The unmatched proteins were searched and annotated by software InterProScan with the sequence alignment method. All proteins were grouped into three major categories: biological processes, cellular components and molecular functions.

For pathway annotation, Kyoto Encyclopedia of Genes and Genomes (KEGG) online tool was used to describe each identified protein’s metabolic classification. All identified proteins were mapped on the KEGG metabolic pathways by the KEGG online service software “KEGG mapper” and annotated by KEGG online software “KAAS”. Wolfpsort was applied to predict the subcellular localization of each identified protein [20].

### Quantitative real-time PCR validation

Total RNA was extracted using a TRIzol Kit according to the manufacturer’s protocol (Promega, Beijing, China). Residual DNA contamination was removed by RNase-free DNase I (TaKaRa, Dalian, China). QRT-PCR was performed using the SYBR Premix Ex Taq Kit (TaKaRa, Dalian, China) and an ABI PRISM 7700 DNA Sequence Detection System (Applied Biosystems, Shanghai, China). The primer sequences were designed using Primer Premier 5 software (Premier Biosoft International, Palo Alto, CA, USA) (Additional file 1: Table S1). *Actin* gene was used as an internal standard to calculate relative fold-differences based on comparative cycle threshold (2^−ΔΔ*Ct*^) values. The qRT-PCR procedure was as follows: 1 μL of a 1/10 dilution of cDNA in H_2_O was added to 5 μL of 2× SYBR® Green buffer, with 0.1 μM of each primer and H_2_O to a final volume of 10 μL. The reactions were run as follows: 50 °C for 2 min and 95 °C for 10 min, followed by 40 cycles of 95 °C for 30 s, 56 °C for 30 s and 72 °C for 30 s in 96-well optical reaction plates.

### Statistical analysis

For GO or KEGG category, a two-tailed Fisher’s exact test was performed to test the enrichments of the DAPs in all identified proteins. A FDR control method was used to adjust multiple hypothesis testing [21]. A GO or KEGG term with a *P* value < 0.05 was thought significant. The proteins which have TMT intensity values were seen as quantified, and the minimal PIF was set as 0.75. Statistical analyses were carried out using SPSS ver. 19.0 (SPSS Inc. Chicago, US), and a one-way analysis of variance (ANOVA) was used to analyze the expression difference of each protein between different sample groups. Experiments were performed in three biological replicates.

## Results

### Quantitative proteomic analysis

**Fig. 1:**
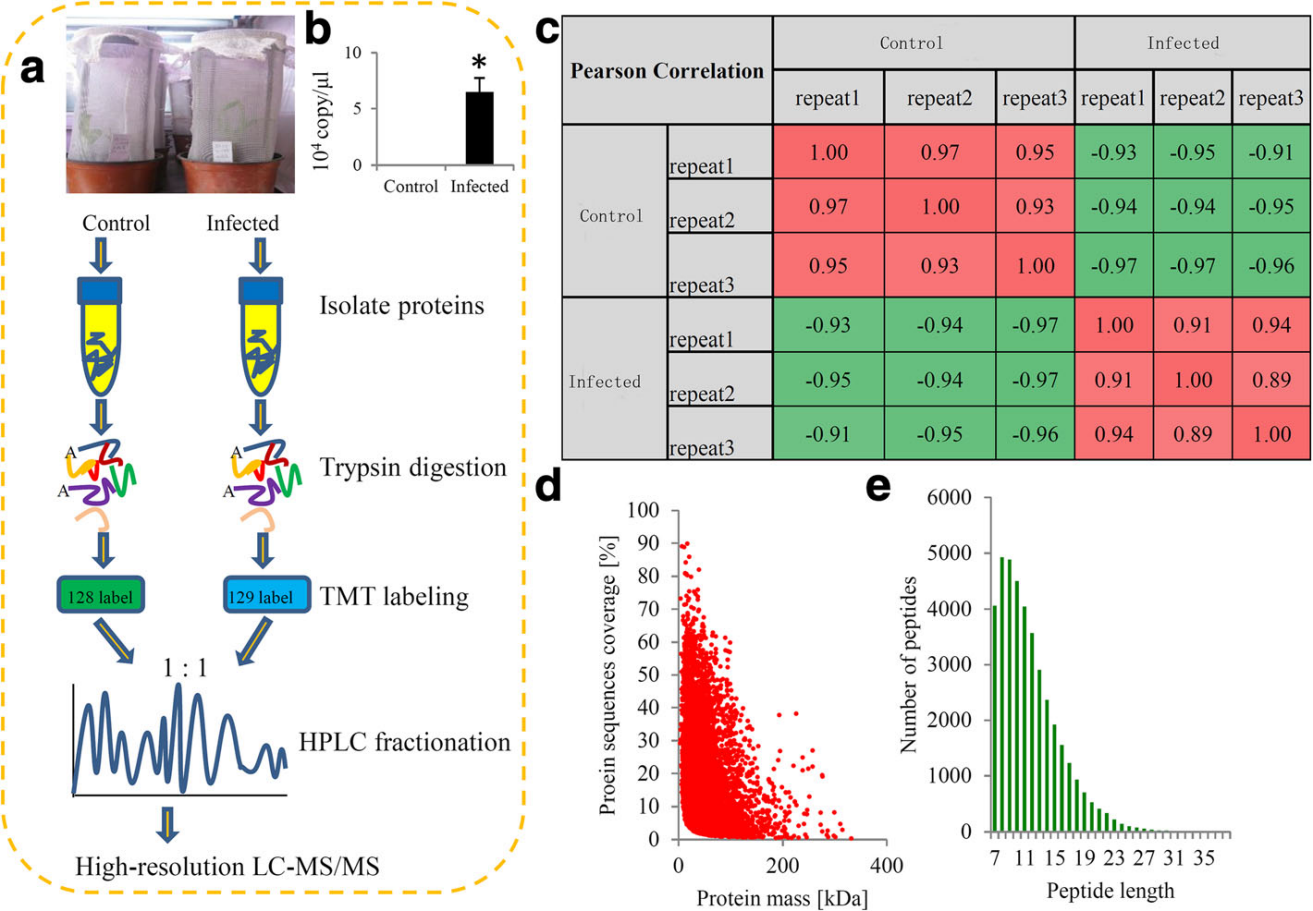
Experimental strategy for quantitative proteome analysis and quality control validation of MS data. **a** Protein were extracted in three biological replicates for each sample group. All protein samples were trypsin digested and analyzed by HPLC-MS/MS. **b** Pearson’s correlation of protein quantitation. **c** Mass delta of all identified peptides. **d** Length distribution of all identified peptides. 128 label: TMT-128 Label Reagent; and 129-label: TMT-129 Label Reagent (ThermoFisher Scientific, Shanghai, China)

An integrated approach involving LC-MS/MS and TMT labeling was applied to analyze the proteomic changes between the control and RBSDV infected maize seedlings. The general workflow is shown in Fig. 1a. No copy of RBSDV was detected in the control seedlings, while approximately 65,000 copies/μL of the S6 segment of RBSDV genome were detected in the infected seedlings (Fig. 1b). Pair-wise Pearson’s correlation coefficients displayed sufficient reproducibility of this experiment (Fig. 1c). After quality validation, 39,614 peptides were detected, and the average mass error was < 0.02 Da, indicating a high mass accuracy of the MS data (Fig. 1d). The lengths of most identified peptides were 7 to 20 amino acid residues, suggesting that our sampling met the required standard (Fig. 1e). The detail information of identified peptides, including peptides sequences, matching scores, precursor charges, modifications, delta mass, has been added to Additional file 2: Table S2.

A total of 7615 proteins were identified, 6319 of which were quantified. To further understand their functions, all identified proteins were annotated according to different categories, including GO terms, predicted functional domains, KEGG pathways, and subcellular localizations. The detailed information of all identified proteins is listed in Additional file 3: Table S3.

### Impacts of RBSDV infection on the global proteome of maize seedlings

Among the quantified proteins, 116 proteins were identified as DAPs between the infected seedlings and control seedlings (Additional file 4: Table S4). Six proteins, such as a dehydrin (C4J477), a patatin (C0HDU5), a kinesin-like protein (K7V9G8), a heat shock protein (A0A0B4J2X0), a F1F0-ATPase inhibitor protein (B6TGD4), and a Glycine-rich RNA binding protein (A0A0B4J3D6), were up-regulated over 1.5-fold by RBSDV infection compared with the control. Five proteins, including a phosphoethanolamine N-methyltransferase (A7XZC6), a manganese transport protein-like protein (A0A096QZB3), a probable 5′-adenylylsulfate reductase 1 (A0A096RBY4), a putative glucose-6-phosphate/phosphate-translocator (A0A0B4J2Y0), and O-methyltransferase ZRP4 (K7UX80), were down-regulated over 1.5-fold by RBSDV infection compared with the control.

**Fig. 2:**
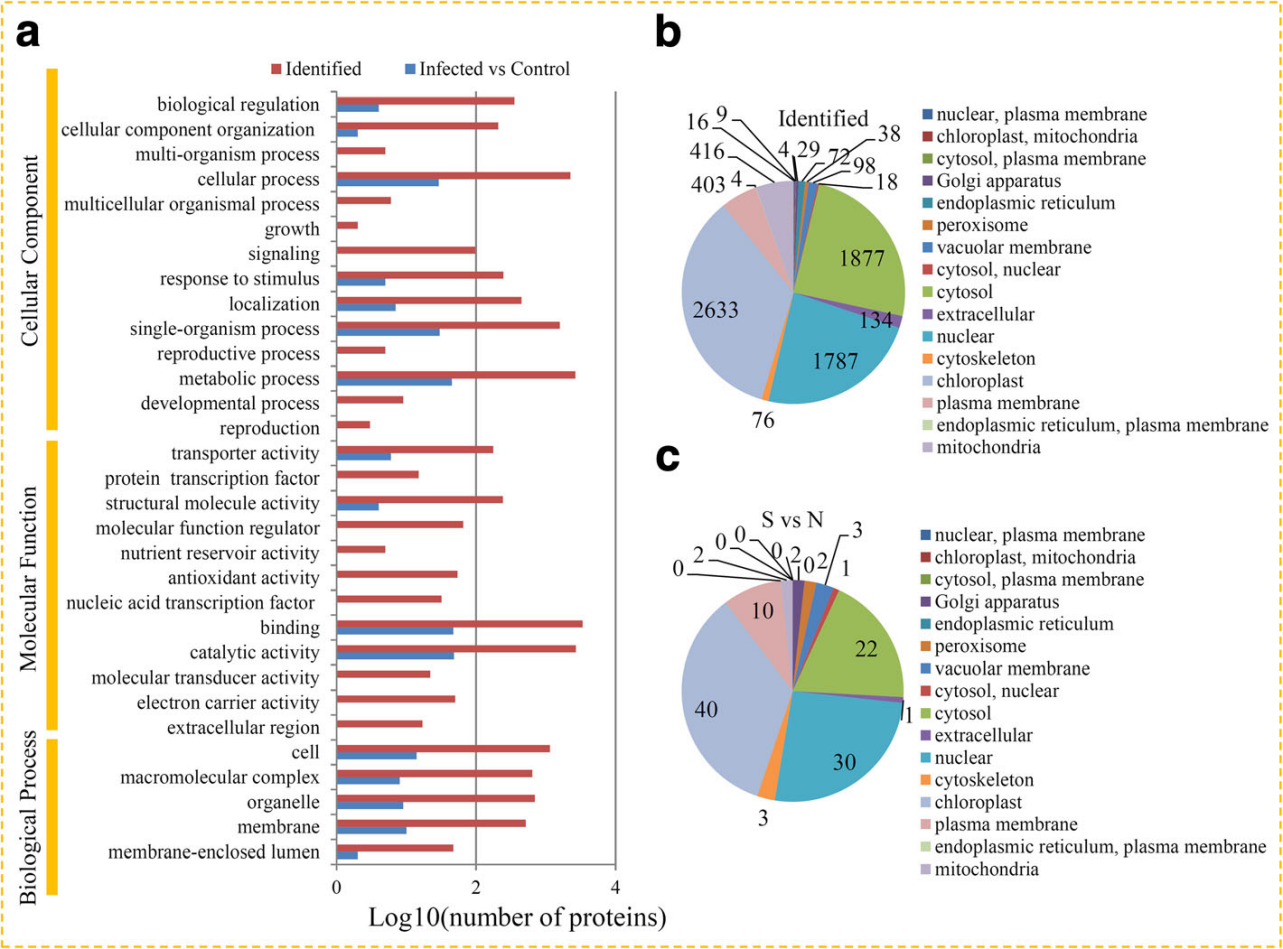
Classification of all identified proteins and DAPs. **a** GO analysis of all identified proteins and DAPs. All proteins were classified by GO terms based on their cellular component, molecular function, and biological process. Subcellular locations of identified proteins (**b**) and differential accumulated proteins (**c**)

The GOs representing all identified proteins and the DAPs under RBSDV infection were grouped into different categories (Fig. 2a). In the biological process category, 2643 identified proteins and 45 DAPs were involved in “metabolic process”, 2255 identified proteins and 29 DAPs were involved in “cellular process”, and 1590 identified proteins and 30 DAPs were involved in “single-organism process”. In the molecular function category, 2691 identified proteins and 48 DAPs had “catalytic activities”, 3373 identified proteins and 47 DAPs had “binding” activities, and 176 identified proteins and 6 DAPs had “transporter activities”. In the cellular components category, 1145 identified proteins and 14 DAPs were “cell”-related proteins, 514 identified proteins and 10 DAPs were “membrane”-related proteins, and 697 identified proteins and 9 DAPs were “organelle”-related proteins.

All identified proteins and the DAPs were grouped based on their subcellular localizations. For identified proteins, 16 subcellular components were identified, including 2633 chloroplast-localized proteins, 1877 cytosol-localized proteins, and 1787 nuclear-localized proteins (Fig. 2b). For the DAPs, only 11 subcellular components were recognized, including 40 chloroplast-localized DAPs, 22 cytosol-localized DAPs, and 30 nuclear-localized DAPs (Fig. 2c).

### Enrichment analysis of DAPs under RBSDV infection

**Fig. 3:**
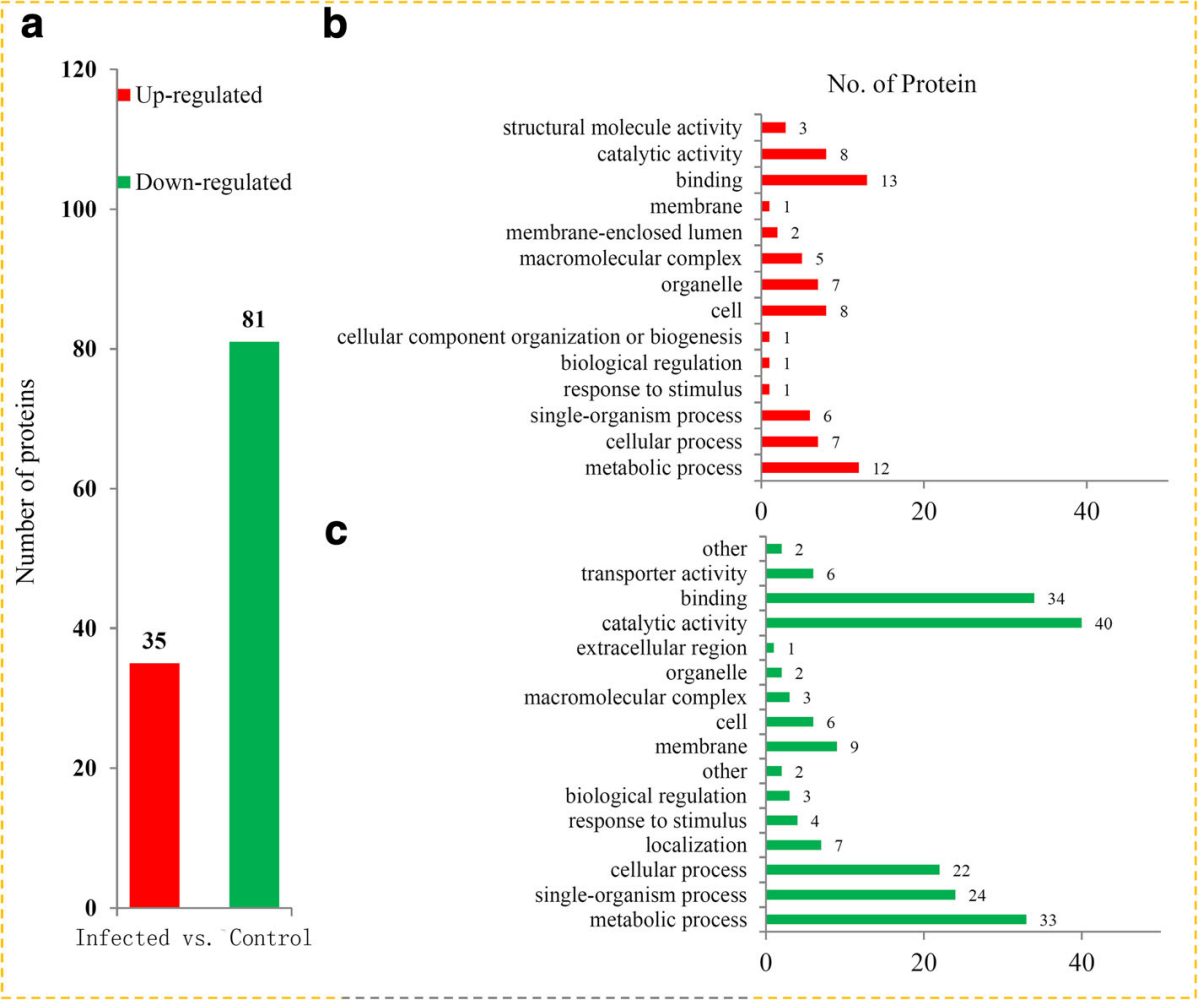
Variations in protein levels between the infected and control seedlings of maize. **a** The numbers of up-regulated proteins and down-regulated proteins in the infected seedlings compared to the control seedlings. Distribution of the up-regulated proteins (**b**) and down-regulated proteins (**c**) with GO annotation

Among the DAPs, 35 proteins were significantly up-regulated and 81 proteins were significantly down-regulated (Fig. 3a). A majority of the up-regulated proteins were mainly associated with ‘binding’ (13 proteins), ‘metabolic processes’ (12 proteins) and ‘cell structure’ (8 proteins) (Fig. 3b). For down-regulated proteins, the top five GO terms were ‘catalytic activity’ (40 proteins), ‘binding’ (34 proteins), ‘metabolic processes’ (33 proteins), ‘single-organism processes’ (24 proteins) and ‘cellular processes’ (22 proteins) (Fig. 3c).

**Fig. 4:**
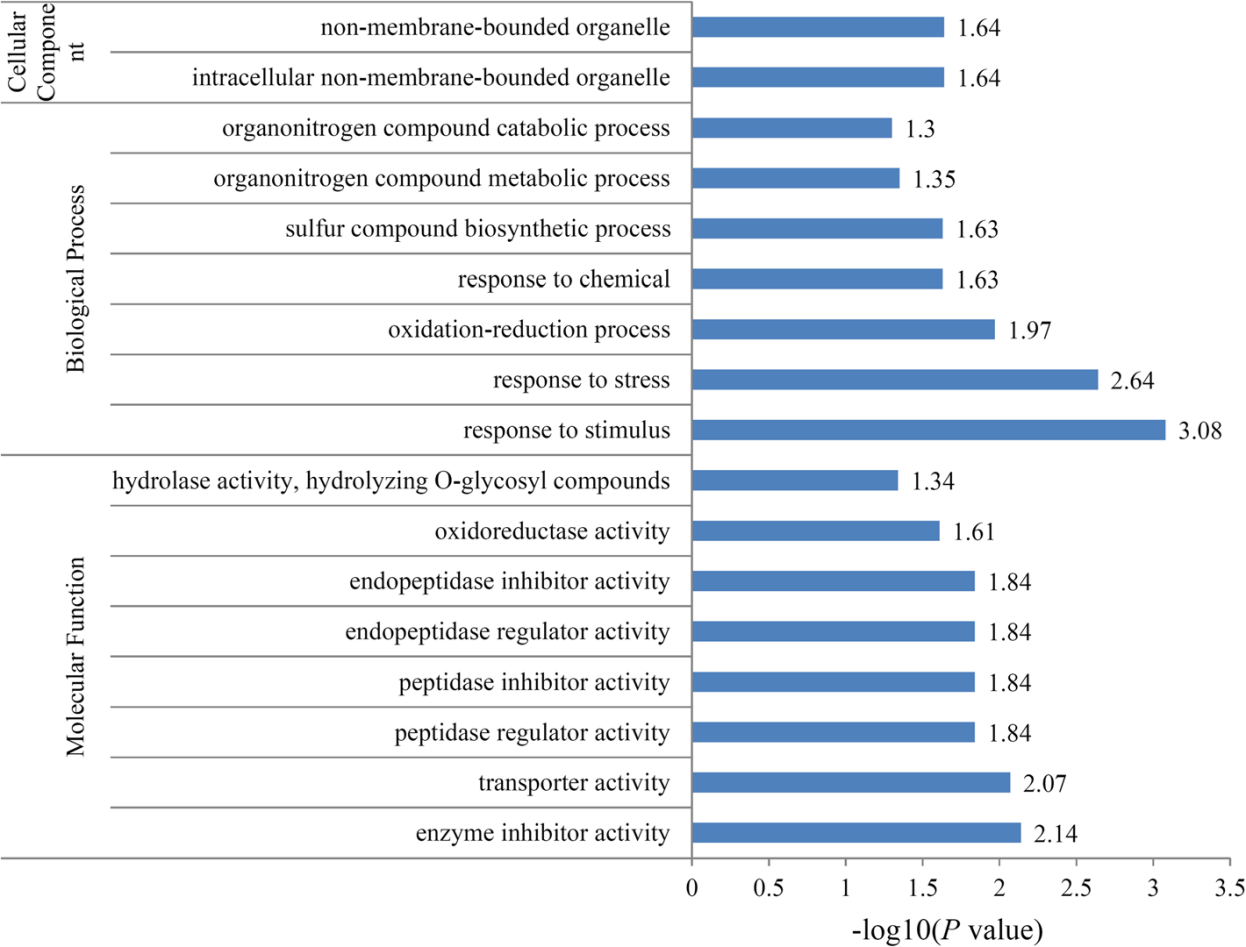
Enrichment analysis of the DAPs in maize during *RBSDV* infection. Significantly enriched GO terms of the DAPs concerning cellular component, molecular function and biological process

The biological functions of the DAPs could also be identified by their GO annotations. The quantified proteins from the four categories were plotted for GO enrichment-based cluster analysis. For the ‘Molecular Function’ category, the DAPs were enriched in ‘enzyme inhibitor activity’, ‘transporter activity’, ‘peptidase regulator activity’, ‘peptidase inhibitor activity’, ‘endopeptidase regulator activity’, ‘endopeptidase inhibitor activity’, ‘oxidoreductase activity’, ‘hydrolase activity, hydrolyzing O-glycosyl compounds’ terms; for the ‘Cellular Component’ category, the DAPs were enriched in ‘intracellular non-membrane-bounded organelle’, ‘non-membrane-bounded organelle’ terms; For the ‘Biological Process’ category, the DAPs were enriched in several GO terms, such as ‘response to stimulus’, ‘response to stress’, ‘oxidation-reduction process’, ‘response to chemical’, ‘sulfur compound biosynthetic process’, ‘organonitrogen compound metabolic process’, ‘organonitrogen compound catabolic process’ (Fig. 4).

**Fig. 5:**
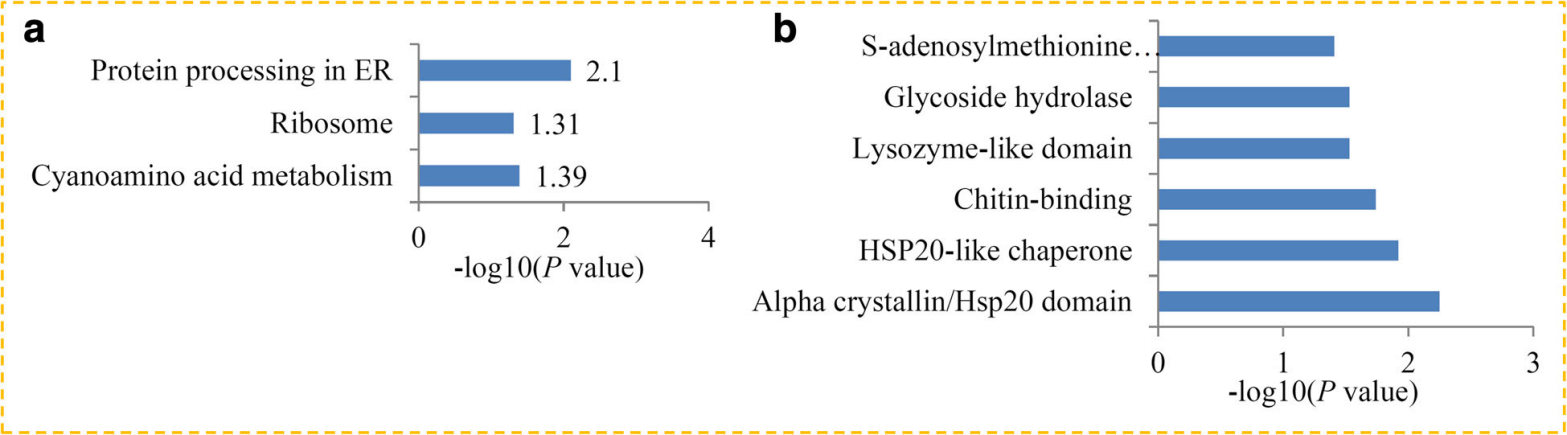
KEGG and domain enrichment analysis of the DAPs in maize during RBSDV infection. **a** Significantly enriched KEGG terms of the DAPs. **b** Significantly enriched protein domains of the DAPs

KEGG enrichment analysis revealed that the DAPs were most strongly associated with ‘cyanoamino acid metabolism’, ‘protein processing in ER’, and ‘ribosome’ pathways (Fig. 5a). Protein domain enrichment analysis revealed that six protein domains, including ‘S-adenosylmethionine synthetase’,‘HSP20-like chaperone’, ‘α-Crystallin’, ‘Glycoside hydrolase’, ‘Lysozyme-like domain’ and ‘Chitin-binding’, were enriched in the DAPs (Fig. 5b).

### Identification of the DAPs involved in metabolic pathways

In our study, 15 DAPs in the RBSDV-infected maize were identified to be involved in six metabolic pathways. In the ‘isoquinoline alkaloid biosynthesis pathway’, one protein (K7TGW6) was significantly up-regulated by RBSDV infection. In the ‘sulfur metabolism’ pathway, two proteins (A0A096RBY4 and C0PFQ7) were significantly down-regulated by RBSDV infection. In the ‘glycerophospholipid metabolism’ pathway, two proteins (B4FKD4 and A7XZC6) were significantly reduced by RBSDV infection. In the ‘Glyoxylate and dicarboxylate metabolism’ pathway, two proteins (B7ZYT6 and K7U1Y7) were significantly reduced by RBSDV infection. In the ‘biosynthesis of amino acids’ pathway, one significantly induced protein (C0P3B4) and five significantly reduced proteins (K7VBU7, K7VC35, B8A068, B6SS03 and K7U2E4) were identified and in the ‘purine metabolism’ pathway, one significantly induced protein (K7TPZ5) and one significantly reduced protein (K7UGQ5) were identified (Table 1).

**Table 1:** Identification of the DEPs involved in metabolic pathways

Protein accession	Gene name	Protein description	MW [kDa]	Infected/Normal Ratio	Infected/Normal *P* value
Isoquinoline alkaloid biosynthesis					
K7TGW6	Zm.92805	Polyphenol oxidase family protein	67.066	1.349774521	0.035047423
Sulfur metabolism					
A0A096RBY4	aprl2	5′-Adenylylsulfate reductase 1	49.526	0.561497326	0.017965114
C0PFQ7	Zm.95122	Bifunctional 3′-phosphoadenosine 5′-phosphosulfate synthethase	53.78	0.793914246	0.017550865
Glycerophospholipid metabolism					
B4FKD4	LOC100283662	Acyl-protein thioesterase 2	26.847	0.777615216	0.036845308
A7XZC6	LOC100383478	Phosphoethanolamine N-methyltransferase	56.774	0.640561507	0.019112186
Glyoxylate and dicarboxylate metabolism					
B7ZYT6	LOC100279574	Ribulose bisphosphate carboxylase small chain	17.859	0.803660566	0.00348751
K7U1Y7	Zm.133422	Glyoxylate/hydroxypyuvate reductase HPR3	35.576	0.78506559	0.035887012
Biosynthesis of amino acids					
C0P3B4	Zm.21713	Phosphoglycerate mutase-like protein	26.235	1.31338265	0.001880484
K7VBU7	Zm.85396	D-3-phosphoglycerate dehydrogenase	48.914	0.791407019	0.025750889
K7VC35	Zm.66568	S-adenosylmethionine synthethase	42.54	0.770235935	0.025440529
B8A068	Zm.20018	S-adenosylmethionine synthethase	43.072	0.757097792	0.012769647
B6SS03	Zm.155641	Arogenate dehydrogenase isoform 2	38.561	0.77589852	0.023596168
K7U2E4	Zm.24636	Amine oxidase	82.989	0.779698303	0.04484565
Purine metabolism					
K7TPZ5	Zm.138895	Aminoimidazolecarboximide ribonucleotide transformylase	65.704	1.222222222	0.0327844
K7UGQ5	LOC100275070	Putative adenine phosphoribosyltransferase form 2	21.25	0.821181647	0.046836087

### Validation of the expression of several RBSDV responsive genes

To verify the differential expression levels of some RBSDV responsive genes, a qRT-PCR assay with independent samples collected from the control and RBSDV-infected seedlings was performed. In total, six key RBSDV responsive genes were randomly selected, including a polyphenol oxidase family protein encoding gene (Zm.92805), a bifunctional 3′-phosphoadenosine 5′-phosphosulfate synthethase encoding gene (Zm.95122), a glyoxylate/hydroxypyuvate reductase 3 encoding gene (Zm.133422), an S-adenosylmethionine synthethase encoding gene (Zm.66568), an amine oxidase encoding gene (Zm.24636), and aminoimidazolecarboximide ribonucleotide transformylase encoding gene (Zm.138895). The expression levels of these selected genes were basically consistent with the proteomic analyses (Additional file 5: Figure S1).

## Discussion

High-throughput proteomic analysis has been developed to reveal the responses of host plants to infection by various plant viruses at the protein level [15, 19, 22]. MRDD, which is caused by RBSDV, is a severe disease that results in substantial yield losses in the Yellow-Huai-Hai River plain of China [23]. In our work, TMT-based proteomic method was used to analyze the alterations in protein abundance between control and RBSDV-infected seedlings. These results will enhance the understanding of regulatory mechanisms involved in maize response to RBSDV infection.

Several studies on the responses of rice to RBSDV infection at protein level have been performed. Approximately 1800 protein spots were detected by 2D-PAGE, out of which, 69 spots were identified as DAPs under long-term RBSDV infection in the susceptible rice cultivar Huai 5 [17]. A label-free quantitative proteomics analysis provided a comprehensive view about the response of RBSDV-infected rice to cytosinpeptidemycin treatment. Several abscisic acid-related proteins were regulated, suggesting that cytosinpeptidemycin may activate the abscisic acid pathway to eliminate RBSDV in rice [24]. In maize, approximately 1200 protein spots were detected and 91 DAPs were identified under RBSDV infection by 2-D method [25]. However, the number of identified proteins is not too much. In our study, 7615 proteins were identified, suggesting a deeper comprehensive analysis of DAPs in maize under RBSDV infection.

In 2013, comparative proteomic analysis revealed the cross-talk between the responses induced by H_2_O_2_ and by long-term RBSDV infection in rice [17]. Siginificantly changes in the photosynthesis-, redox homeostasis-, and carbohydrate metabolism-related proteins were observed in rice rather than in maize. Changes in the amino acid metabolism-, cell wall modification-, and stress-related proteins were observed in both rice and maize.

Plants have evolved several highly efficient and sophisticated strategies to respond to different abiotic stresses, particular in virus infection [26, 27]. GO analysis revealed that five ‘response to stimulus’-related proteins, including one up- and four down-regulated proteins, were significantly altered by RBSDV infection in maize (Fig. 3b and c). In beans, a SKn-type dehydrin gene, *PvSR3*, plays an essential role in the resistance to damage caused by heavy metals [28]. Among these ‘response to stimulus’-related proteins, a dehydrin protein (C4J477) was down-regulated over 1.5-fold by RBSDV infection, suggesting that dehydrin proteins may also play a role in the responses to RBSDV infection. Phytohormones, particularly auxin, participate in the regulation of plant-microbe interactions [29]. In our study, an auxin response factor (ARF, A0A096R641), another ‘response to stimulus’-related protein, was significantly reduced by RBSDV infection. The data indicated that ARF-mediated auxin-signaling may be involved in the response to RBSDV infection. Besides, several studies have reported that there were many interactions between viral proteins and components of the ubiquitin and ubiquitin-like protein pathways [30]. In our study, a number ubiquitin pathway proteins were identified, suggesting an important role of ubiquitin-proteasome degradation in the response to RBSDV infection. Moreover, RNA-binding protein is essential for resistance pathway against viral pathogens [31]. In maize, 31 RNA-binding proteins were identified. Differential accumulated RNA-binding proteins may be involved in host resistance protein-mediated resistance against viral and bacterial pathogens.

Plants are thought to produce specific metabolites to resist pathogen invasion [32, 33]. Transcriptomic analysis of apple leaves showed that the metabolic pathway of cyanoamino acid biosynthesis was activated during *Alternaria alternata* apple pathotype infection [34]. In our study, KEGG enrichment analysis revealed that three pathways, such as the cyanoamino acid metabolism pathway, were enriched under RBSDV infection. Furthermore, 15 DAPs involved in six metabolic pathways were identified in maize under RBSDV infection (Table 1). For example, a polyphenol oxidase family protein (K7TGW6) associated with isoquinoline alkaloid biosynthesis was identified as a significantly up-regulated protein. Plant-like biosynthesis of isoquinoline alkaloids has been discovered in pathogenic bacteria, such as *Aspergillus fumigatus* [35]. Increases in the isoquinoline alkaloid biosynthesis may be required for the establishment of maize-RBSDV interaction. Additionally, two sulfur metabolism-related proteins, including an 5′-adenylylsulfate reductase and a bifunctional 3′-phosphoadenosine 5′-phosphosulfate synthetase, were significantly down-regulated by RBSDV infection in maize. Management of sulfur uptake from the soil contributes to the disease resistance of plants [36]. For example, increasing the absorption of sulfate in *V. dahliae* infected tomato roots enhances tomato resistance to *V. dahliae* [37]. In maize, decreased expression of the two sulfur metabolism-related proteins indicated that sulfur may play a significant role in resistance to RBSDV infection.

Virus-induced alterations in primary metabolism, such as biosynthesis of amino acids and purine metabolism, affect the tolerance of plant to various viruses [38]. There is a close relationship between plasma amino acid profiles and the different stages of hepatitis B infection [39]. In rice plants, however, no significant changes in amino acid content was observed after RBSDV infection [40]. In our study, six proteins involved in biosynthesis of amino acids were identified as DAPs, suggesting that there was a difference in responses to RBSDV infection between rice and maize. Application of allopurinol, an inhibitor of purine metabolism, enhances susceptibility of tobacco plants to Tobacco mosaic virus infection [41]. In maize, two purine metabolism-related proteins were identified as DAPs under RBSDV infection. Our data revealed that various metabolic pathways were involved in the responses of maize to RBSDV infection. Our data may provide useful resource for maize breeding.

## Conclusions

In our study, a proteomic approach was used to investigate DAPs under RBSDV infection in maize seedlings. In total, 116 DAPs were identified and characterized based on their major biological functions. Our data provides fundamental resources for identifying candidate proteins and metabolic pathways involved in the response of maize plants to RBSDV infection.

## Additional files

Additional file 1: **Table S1.** The primer sequences for qRT-PCR. (XLSX 8 kb)

Additional file 2: **Table S2.** The detail information of identified peptides pertinent to detected proteins. (XLSX 8119 kb)

Additional file 3: **Table S3.** The annotation information of all identified proteins. (XLSX 1598 kb)

Additional file 4: **Table S4.** The detail information of DAPs. (XLSX 40 kb)

Additional file 5: **Figure S1.** Validation of the expression of several RBSDV responsive genes. (DOCX 20 kb)

## Abbreviations

ACN: Acetonitrile
ANOVA: Analysis of variance
CAMTA: Calmodulin-binding transcription activator
DAP: Differentially accumulated protein
DEG: Differential expressed gene
FA: Formic acid
FDR: False discovery rate
GO: Gene Ontology
HPLC: High Performance Liquid Chromatography
KEGG: Kyoto Encyclopedia of Genes and Genomes
MRDD: Maize rough dwarf disease
MRDV: Maize rough dwarf virus
MS: Mass spectrometry
RBSDV: Rice black streaked dwarf virus
TMT: Tandem Mass Tag
