# Supplementary material for: Comparative proteomic analysis of maize (Zea mays L.) seedlings under rice black-streaked dwarf virus infection
Source: BMC Plant Biol. 2018 Sep 12;18:191. doi: 10.1186/s12870-018-1419-x (PMC6136180; doi:10.1186/s12870-018-1419-x)
Supplement: Supplementary file 5 — Figure S1. Validation of the expression of several RBSDV responsive genes. (DOCX 20 kb) [file 12870_2018_1419_MOESM5_ESM.docx]

Fig. S1 Real-time quantitative PCR validation of several selected RBSDV responsive genes. The data were analyzed by three independent repeats, and standard deviations were shown with error bars. Signiﬁcant differences in expression level were indicated by “*”.
